# Bis(2,6-dimethyl­pyridinium) dibromo­iodate bromide

**DOI:** 10.1107/S1600536812035702

**Published:** 2012-08-23

**Authors:** Rawhi Al-Far, Basem F. Ali, Salim F. Haddad

**Affiliations:** a Faculty of Science and IT, Al-Balqa’a Applied University, Salt, Jordan; b Department of Chemistry, Al al-Bayt University, Mafraq 25113, Jordan; c Department of Chemistry, The University of Jordan, Amman 11942, Jordan

## Abstract

In the title salt, 2C_7_H_10_N^+^·IBr_2_^−^·Br^−^, each of the anions, *viz.* [IBr_2_]^−^ and Br^−^, lie on a twofold axis. The IBr_2_^−^ anion is almost linear, with a Br—I—Br angle of 178.25 (3)°. The cation is essentially planar (r.m.s. deviation = 0.0067 Å). In the crystal, each Br^−^ anion links two cations *via* N—H⋯Br⋯H—N hydrogen-bonding inter­actions.

## Related literature

For background to this study, see: Kochel (2006 ▶). For comparison bond lengths and angles, see: Gardberg *et al.* (2002 ▶); Ahmadi *et al.* (2008 ▶).
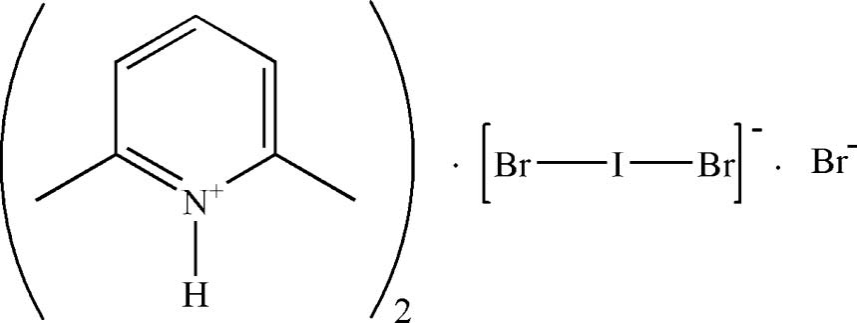


## Experimental

### 

#### Crystal data

2C_7_H_10_N^+^·Br_2_I^−^·Br^−^
*M* *_r_* = 582.92
Monoclinic, 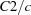

*a* = 13.8627 (16) Å
*b* = 11.3622 (9) Å
*c* = 13.8957 (15) Å
β = 108.885 (13)°
*V* = 2070.9 (4) Å^3^
*Z* = 4
Mo *K*α radiation
μ = 7.33 mm^−1^
*T* = 293 K
0.34 × 0.28 × 0.15 mm

#### Data collection

Agilent Xcalibur Eos diffractometer
Absorption correction: analytical (*CrysAlis PRO*; Agilent, 2011 ▶) *T*_min_ = 0.578, *T*_max_ = 0.733
4417 measured reflections
1834 independent reflections
1280 reflections with *I* > 2σ(*I*)
*R*_int_ = 0.034

#### Refinement

*R*[*F* ^2^ > 2σ(*F* ^2^)] = 0.040
*wR*(*F* ^2^) = 0.104
*S* = 1.05
1834 reflections
92 parameters
H-atom parameters constrained
Δρ_max_ = 0.52 e Å^−3^
Δρ_min_ = −0.58 e Å^−3^

### 

Data collection: *CrysAlis PRO* (Agilent, 2011 ▶); cell refinement: *CrysAlis PRO*; data reduction: *CrysAlis PRO*; program(s) used to solve structure: *SHELXS97* (Sheldrick, 2008 ▶); program(s) used to refine structure: *SHELXL97* (Sheldrick, 2008 ▶); molecular graphics: *SHELXTL* (Sheldrick, 2008 ▶); software used to prepare material for publication: *SHELXTL*.

## Supplementary Material

Crystal structure: contains datablock(s) I, global. DOI: 10.1107/S1600536812035702/pv2580sup1.cif

Structure factors: contains datablock(s) I. DOI: 10.1107/S1600536812035702/pv2580Isup2.hkl

Supplementary material file. DOI: 10.1107/S1600536812035702/pv2580Isup3.cml

Additional supplementary materials:  crystallographic information; 3D view; checkCIF report

## Figures and Tables

**Table 1:** Hydrogen-bond geometry (Å, °)

*D*—H⋯*A*	*D*—H	H⋯*A*	*D*⋯*A*	*D*—H⋯*A*
N1—H1*A*⋯Br2	0.86	2.45	3.315 (5)	179

**Fig. 1.**
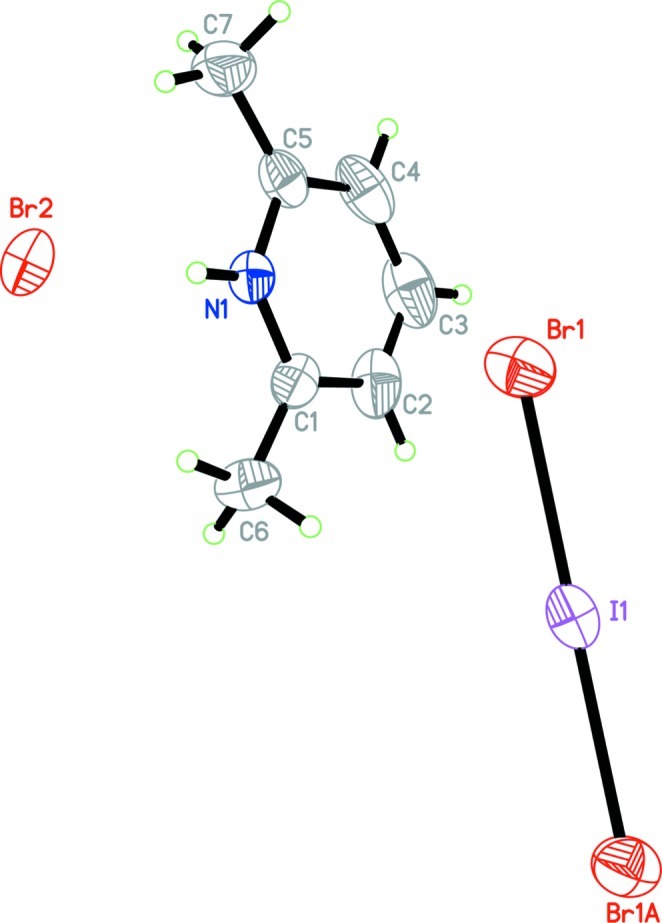
Molecular configuration and atom naming scheme for I. Displacement ellipsoids are drawn at the 30% probability level. A stands for the symmetry operation: -x + 1, y, -z + 1/2

**Fig. 2.**
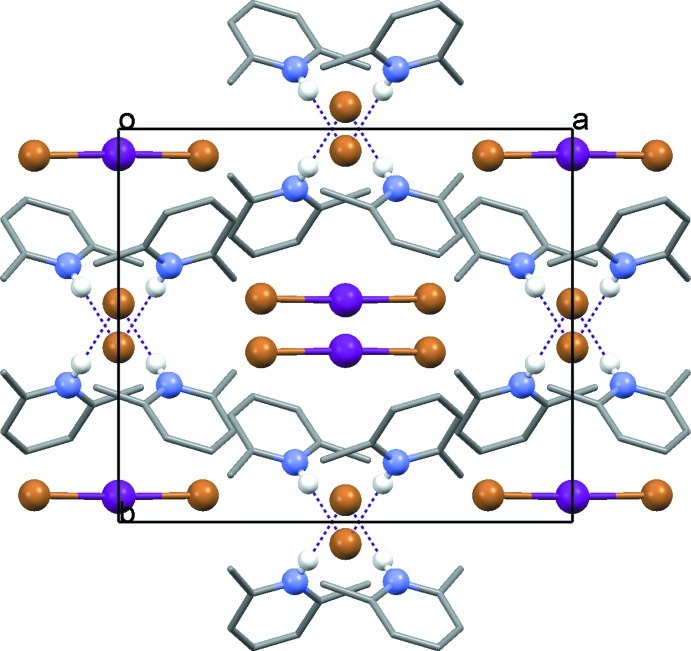
Packing diagram of I, down crystallographic c axis. Interspecies hydrogen bonds are shown as dashed lines.

## supplementary crystallographic information

### Comment

Polyhalides display a variety of structures. Various compounds with interesting structures were found when protonated aromatic nitrogen bases were combined with polyhalides (Kochel, 2006). Herein, we report the crystal structure of [(C_7_H_10_N)^+^]_2_. [IBr_2_] ^–^. Br^-^, (I). Few crystals of (I) were found as an unexpected product from reaction mixture of CdI_2_, HBr, 2,6-dimethylpyridine and Br_2_ upon attempting to formulate [(C_7_H_10_N)]_2_ [CdBr_4_] salt of 2,6-dimethylpyrinium.

The title salt is depicted in Fig. 1. The IBr_2_^–^ anion is symmetrical and almost linear, Br1—I—Br1^i^ angle of 178.25 (3) °; (i) –*x* + 1, *y*, -*z* + 1/2], with I—Br1 distance of 2.7117 (9) Å. These values are in agreement with the values reported in the literature (Gardberg *et al.*, 2002). The molecular dimensions of the cation are as expected (Ahmadi *et al.*, 2008).

The cations are arranged as zigzag stacks parallel to the *c*-axis (Fig. 2). Moreover, alternating Br^-^ and IBr_2_^-^ anions form stacks that separate the cations. Each bromide anion is hydrogen bonded *via* N1—H1A···Br2 with two cations along the *b*-axis (Table 1). There are no significant Br···Br or aryl···aryl interactions in the crystal structure; the shortest Br···Br separation is just greater than 5.0 Å and the shortest distance between the ring centroids is over 4.8 Å.

### Experimental

A solution of CdI_2_ (0.37 g, 1 mmol) dissolved in 95% EtOH (10 ml) and 2 ml 60% HBr solution was added to a mixture of 2,6-dimethylpyridine (0.11 g, 1 mmol) dissolved in 95% EtOH (10 ml), 60% HBr (2 ml) and molecular bromine (2 ml). The resulting mixture was refluxed for 2 hr. On cooling few reddish crystals of the title complex were found mixed in the bulk of the precipitate formed which proved to be mainly 2,6-dimethylpyridinium bromide.

### Refinement

All H atoms were positioned geometrically and refined using a riding model, with N—H = 0.86 Å and C—H = 0.93 and 0.96 Å, for aryl and methyl H-atoms, respectively. The *U*_iso_(H) were allowed at 1.5*U*_eq_(C methyl) or 1.2*U*_eq_(N/C non-methyl).

### Crystal data

**Table:**

2C_7_H_10_N^+^·Br_2_I^−^·Br^−^	*F*(000) = 1104
*M**_r_* = 582.92	*D*_x_ = 1.870 Mg m^−^^3^
Monoclinic, *C*2/*c*	Mo *K*α radiation, λ = 0.71073 Å
Hall symbol: -C 2yc	Cell parameters from 1414 reflections
*a* = 13.8627 (16) Å	θ = 3.0–29.4°
*b* = 11.3622 (9) Å	µ = 7.33 mm^−^^1^
*c* = 13.8957 (15) Å	*T* = 293 K
β = 108.885 (13)°	Block, orange
*V* = 2070.9 (4) Å^3^	0.34 × 0.28 × 0.15 mm
*Z* = 4	

### Data collection

**Table:**

Agilent Xcalibur Eos diffractometer	1834 independent reflections
Radiation source: Enhance (Mo) X-ray Source	1280 reflections with *I* > 2σ(*I*)
Graphite monochromator	*R*_int_ = 0.034
Detector resolution: 16.0534 pixels mm^-1^	θ_max_ = 25.0°, θ_min_ = 3.1°
ω scans	*h* = −16→12
Absorption correction: analytical (*CrysAlis PRO*; Agilent, 2011)	*k* = −12→13
*T*_min_ = 0.578, *T*_max_ = 0.733	*l* = −16→16
4417 measured reflections	

### Refinement

**Table:**

Refinement on *F*^2^	Primary atom site location: structure-invariant direct methods
Least-squares matrix: full	Secondary atom site location: difference Fourier map
*R*[*F*^2^ > 2σ(*F*^2^)] = 0.040	Hydrogen site location: inferred from neighbouring sites
*wR*(*F*^2^) = 0.104	H-atom parameters constrained
*S* = 1.05	*w* = 1/[σ^2^(*F*_o_^2^) + (0.0423*P*)^2^ + 1.4129*P*] where *P* = (*F*_o_^2^ + 2*F*_c_^2^)/3
1834 reflections	(Δ/σ)_max_ = 0.001
92 parameters	Δρ_max_ = 0.52 e Å^−^^3^
0 restraints	Δρ_min_ = −0.58 e Å^−^^3^

### Special details

**Table:**

Geometry. All esds (except the esd in the dihedral angle between two l.s. planes) are estimated using the full covariance matrix. The cell esds are taken into account individually in the estimation of esds in distances, angles and torsion angles; correlations between esds in cell parameters are only used when they are defined by crystal symmetry. An approximate (isotropic) treatment of cell esds is used for estimating esds involving l.s. planes.
Refinement. Refinement of *F*^2^ against ALL reflections. The weighted *R*-factor *wR* and goodness of fit *S* are based on *F*^2^, conventional *R*-factors *R* are based on *F*, with *F* set to zero for negative *F*^2^. The threshold expression of *F*^2^ > σ(*F*^2^) is used only for calculating *R*-factors(gt) *etc*. and is not relevant to the choice of reflections for refinement. *R*-factors based on *F*^2^ are statistically about twice as large as those based on *F*, and *R*- factors based on ALL data will be even larger.

### Fractional atomic coordinates and isotropic or equivalent isotropic displacement parameters (Å^2^)

**Table:**

	*x*	*y*	*z*	*U*_iso_*/*U*_eq_	
I1	0.5000	0.56444 (5)	0.2500	0.0633 (2)	
Br1	0.31439 (6)	0.56808 (6)	0.10417 (6)	0.0846 (3)	
Br2	0.0000	0.55587 (7)	0.2500	0.0665 (3)	
N1	0.1116 (3)	0.3483 (4)	0.1556 (3)	0.0529 (11)	
H1A	0.0824	0.4027	0.1793	0.064*	
C6	0.2531 (5)	0.3799 (6)	0.3092 (5)	0.081 (2)	
H6A	0.2058	0.4369	0.3186	0.122*	
H6B	0.3145	0.4188	0.3090	0.122*	
H6C	0.2688	0.3237	0.3637	0.122*	
C1	0.2069 (5)	0.3183 (5)	0.2109 (5)	0.0614 (16)	
C5	0.0574 (5)	0.2992 (5)	0.0649 (5)	0.0654 (17)	
C2	0.2529 (6)	0.2305 (6)	0.1722 (6)	0.085 (2)	
H2A	0.3183	0.2057	0.2091	0.101*	
C7	−0.0487 (5)	0.3421 (7)	0.0149 (5)	0.093 (2)	
H7A	−0.0643	0.4021	0.0563	0.140*	
H7B	−0.0955	0.2778	0.0069	0.140*	
H7C	−0.0544	0.3742	−0.0506	0.140*	
C4	0.1058 (8)	0.2137 (6)	0.0278 (6)	0.090 (2)	
H4A	0.0720	0.1780	−0.0341	0.108*	
C3	0.2038 (8)	0.1805 (6)	0.0814 (7)	0.098 (3)	
H3A	0.2363	0.1235	0.0550	0.117*	

### Atomic displacement parameters (Å^2^)

**Table:**

	*U*^11^	*U*^22^	*U*^33^	*U*^12^	*U*^13^	*U*^23^
I1	0.0866 (5)	0.0525 (4)	0.0601 (4)	0.000	0.0366 (3)	0.000
Br1	0.0928 (6)	0.0837 (5)	0.0703 (5)	0.0069 (4)	0.0166 (4)	−0.0037 (4)
Br2	0.0573 (5)	0.0564 (5)	0.0949 (7)	0.000	0.0371 (5)	0.000
N1	0.059 (3)	0.046 (3)	0.059 (3)	0.006 (2)	0.026 (2)	0.005 (2)
C6	0.065 (4)	0.083 (5)	0.082 (5)	0.005 (4)	0.005 (4)	−0.001 (4)
C1	0.058 (4)	0.056 (4)	0.074 (4)	0.010 (3)	0.028 (3)	0.023 (3)
C5	0.088 (5)	0.058 (4)	0.058 (4)	−0.014 (4)	0.034 (4)	−0.002 (3)
C2	0.098 (6)	0.068 (5)	0.107 (6)	0.035 (4)	0.061 (5)	0.032 (4)
C7	0.076 (5)	0.116 (6)	0.078 (5)	−0.017 (5)	0.012 (4)	0.001 (4)
C4	0.152 (8)	0.063 (5)	0.068 (5)	−0.018 (5)	0.054 (5)	−0.014 (4)
C3	0.151 (8)	0.066 (5)	0.101 (6)	0.031 (5)	0.076 (6)	0.011 (5)

### Geometric parameters (Å, º)

**Table:**

I1—Br1	2.7117 (9)	C1—C2	1.383 (9)
I1—Br1^i^	2.7117 (9)	C5—C4	1.372 (9)
Br2—Br2^ii^	0.0000	C5—C7	1.490 (9)
Br2—Br2	0.0000	C2—C3	1.350 (10)
N1—C1	1.340 (7)	C2—H2A	0.9300
N1—C5	1.361 (7)	C7—H7A	0.9600
N1—H1A	0.8600	C7—H7B	0.9600
C6—C1	1.483 (8)	C7—H7C	0.9600
C6—H6A	0.9600	C4—C3	1.373 (10)
C6—H6B	0.9600	C4—H4A	0.9300
C6—H6C	0.9600	C3—H3A	0.9300
			
Br1—I1—Br1^i^	178.25 (3)	C4—C5—C7	125.8 (7)
Br2^ii^—Br2—Br2	0 (10)	C3—C2—C1	120.7 (7)
C1—N1—C5	125.0 (5)	C3—C2—H2A	119.6
C1—N1—H1A	117.5	C1—C2—H2A	119.6
C5—N1—H1A	117.5	C5—C7—H7A	109.5
C1—C6—H6A	109.5	C5—C7—H7B	109.5
C1—C6—H6B	109.5	H7A—C7—H7B	109.5
H6A—C6—H6B	109.5	C5—C7—H7C	109.5
C1—C6—H6C	109.5	H7A—C7—H7C	109.5
H6A—C6—H6C	109.5	H7B—C7—H7C	109.5
H6B—C6—H6C	109.5	C5—C4—C3	120.6 (7)
N1—C1—C2	117.0 (6)	C5—C4—H4A	119.7
N1—C1—C6	117.4 (5)	C3—C4—H4A	119.7
C2—C1—C6	125.6 (6)	C2—C3—C4	120.1 (7)
N1—C5—C4	116.6 (6)	C2—C3—H3A	119.9
N1—C5—C7	117.6 (6)	C4—C3—H3A	119.9
			
C5—N1—C1—C2	−0.2 (9)	C6—C1—C2—C3	−179.9 (7)
C5—N1—C1—C6	−178.7 (5)	N1—C5—C4—C3	0.4 (9)
C1—N1—C5—C4	−0.9 (9)	C7—C5—C4—C3	−179.4 (7)
C1—N1—C5—C7	178.9 (5)	C1—C2—C3—C4	−2.2 (11)
N1—C1—C2—C3	1.8 (9)	C5—C4—C3—C2	1.1 (11)

Symmetry codes: (i) −*x*+1, *y*, −*z*+1/2; (ii) −*x*, *y*, −*z*+1/2.

### Hydrogen-bond geometry (Å, º)

**Table:**

*D*—H···*A*	*D*—H	H···*A*	*D*···*A*	*D*—H···*A*
N1—H1*A*···Br2	0.86	2.45	3.315 (5)	179
